# “What I wanted to do was build myself back up and prepare”: qualitative findings from the PERCEPT trial of prehabilitation during autologous stem cell transplantation in myeloma

**DOI:** 10.1186/s12885-023-10799-1

**Published:** 2023-04-17

**Authors:** Orla McCourt, Abigail Fisher, Joanne Land, Gita Ramdharry, Anna L Roberts, Georgios Bekris, Kwee Yong

**Affiliations:** 1 Therapies & Rehabilitation, Inpatient Therapy Office, University College London Hospitals NHS Foundation Trust, University College Hospital, T-1/Lower Ground Floor, 235 Euston Road, London, NW1 2BU UK; 2 Research Department of Haematology, UCL Cancer Institute, University College London, London, UK; 3 UCL Department of Behavioural Science and Health, University College London, London, UK; 4 Queens Square Centre for Neuromuscular diseases, National Hospital for Neurology & Neurosurgery, UCL Institute of Neurology, UCLH NHS Trust, University College London, London, UK

**Keywords:** Haematological cancer, Myeloma, Autologous stem cell transplant, Cancer rehabilitation, Prehabilitation, Physiotherapy, Exercise, Qualitative research, Recruitment, Research participation

## Abstract

**Background:**

The addition of qualitative methodology to randomised controlled trials evaluating complex interventions allows better understanding of contextualised factors and their potential influence on trial delivery and outcomes, as well as opportunities for feedback on trial participation to improve future trial protocols. This study explored the experiences of participation in cancer rehabilitation research during active cancer treatment. Participants were people living with haematological cancer myeloma, undergoing autologous stem cell transplantation (ASCT) recruited to the PERCEPT myeloma pilot trial.

**Methods:**

A qualitative semi-structured interview study, embedded within a pilot randomised controlled trial of a physiotherapist-led exercise intervention delivered before, during and after ASCT among people living with myeloma. Transcripts were analysed using reflexive thematic analysis.

**Results:**

Interviews from 16 trial participants (n = 8 intervention group; n = 8 control group; mean age 61 years, 56% male) were analysed. Four main themes were identified: (1) “It’s not just beneficial for me, it’s for people after me as well”; (2) Disparities in experience of recovery – expectations, feeling prepared and support; (3) “What I wanted to do was build myself back up and prepare”; (4) Active ingredients – participants’ experience of the trial intervention. Participants reported both altruistic and perceived personal gain as motivators for enrolling in the trial. Disappointment caused by allocation to control arm may have led to participants seeking exercise elsewhere, indicating possible contamination of control condition. Disparities in experience of recovery from transplant were evident with intervention participants reporting greater trajectory of recovery.

**Conclusions:**

The findings from this embedded qualitative study highlight numerous considerations required when designing pilot and efficacy trials of complex interventions. The addition of qualitative investigation offers greater understanding of motivations for participation, intervention mechanisms at play as well as effects of participation that may impact interpretation of quantitative outcomes.

**Trial registration:**

Qualitative findings from a prospectively registered pilot trial (ISRCTN15875290), registered 13/02/2019.

**Supplementary Information:**

The online version contains supplementary material available at 10.1186/s12885-023-10799-1.

## Background

Rehabilitation interventions are considered complex interventions. Factors of importance need to consider more than the target behaviour and quantification of exposure to the intervention in terms of dose or number of sessions. The mechanisms through which the intervention is delivered, including to whom, by whom, in what settings, the skills and expertise of the deliverer, also need consideration [1]. Generally, randomised controlled trials (RCT) are considered the most rigorous approach to assessing the efficacy of rehabilitation and exercise interventions, but given their complexity, an entirely quantitative approach to evaluation is no longer considered sufficient for evaluation [2]. The addition of qualitative investigation, as an element of process evaluation, is necessary to better understand and report contextualised factors and their potential influence on the delivery and outcome related to an intervention [1, 3].

Qualitative investigation as part of pilot RCTs is important yet underutilised [4]. In addition to informing better understanding of the intervention and the context in which it acts, qualitative exploration of pilot trial participants may uncover unintended consequences not considered during research protocol development. One example is ‘research participation effect’; a construct proposed to inform better understanding of participant-related biases that may be at play in behavioural intervention research that might not be prevented through the process of randomisation. The concept of research participation effects has evolved from the established notion of understanding that research participants may be influenced by being studied, whether intentionally or subconsciously, historically referred to as the Hawthorne effect [5]. Research participation effects can manifest as early as during approach for consideration of research participation. Therefore, exploring participant interactions with pre-randomisation research activities, as well as assessment activities that may relate to the intervention behaviour (e.g., wearing an activity tracer or completing a log sheet), is necessary as they may have potential to induce unintended behaviour change in both allocation groups [5].

The process of randomisation for allocation in trials assumes that participants, as well as researchers, accept the process from a position of equipoise. However participant preference for allocation, particularly in unblinded trials, is common and can affect continued participation and have implications for validity [6]. Response to allocation in unblinded trials can additionally induce research participation effects or biases post-randomisation, most notably within control participants who may be dissatisfied with allocation [7].

Myeloma is an incurable relapsing-remitting haematological cancer. The repetitive pattern of active disease, remission and relapse results in diminishing disease prognosis and everchanging management approaches [8]. Autologous stem cell transplantation (ASCT) enables use of high-dose chemotherapy to consolidate therapy and is most frequently used as first line therapy in people living with myeloma deemed ‘fit’ enough for intensive anti-cancer therapy [9]. A unique feature of myeloma is disease-related bone destruction and consequent increased risk of fractures. Over a third of people living with myeloma who undergo ASCT will experience high symptom burden for up to nine months post-ASCT [10]. Qualitative literature supports the positive experience of increased physical activity (PA) resulting in proportional gains in perceived physical vigour and strength among people living with myeloma undergoing ASCT [11]. A thematic synthesis of qualitative literature from this population found ‘exercise for recovery’ to be a key analytical theme of high confidence [12]. Quantitative studies also indicate that more physically active myeloma survivors experience greater quality of life (QOL), less side-effects related to treatment and less fatigue compared to less active counterparts [13, 14]. Exercise intervention trials in myeloma have demonstrated that exercise is safe with indication of positive effects on physical, psychological and QOL outcomes [15, 16]. However, definitive efficacy has not yet been established due to small, underpowered trial designs and heterogeneity in intervention and outcome measurement [17].

PERCEPT was a two-arm pilot RCT of physiotherapist-led exercise prehabilitation and rehabilitation intervention for people living with myeloma undergoing ASCT [18, 19]. The pilot trial indicated that delivering prehabilitation during ASCT is feasible and safe for people living with myeloma. Of 50 participants recruited, 33 (66%) completed a final study assessment. Five (10%) participants were lost to follow-up, two (4%) participants died and 20% (n = 10) of participants were withdrawn due to not proceeding to ASCT because of progression of disease or other clinical decision. Secondary outcomes showed promise for the exercise intervention with improvements in QOL, fatigue, functional capacity and PA evident pre-ASCT and 3 months post-ASCT [20]. The aim of this qualitative study was to explore the experiences of participants who took part in the PERCEPT myeloma pilot trial in order to aid the design of a fully powered RCT.

## Materials and methods

### Design and setting

This qualitative study was undertaken as part of the PERCEPT trial, a pilot RCT of an exercise prehabilitation and rehabilitation exercise intervention delivered as part of the ASCT pathway at a UK centre [18, 19]. The trial was prospectively registered (ISRCTN 15875290). Ethical approval was obtained (London – Camden & Kings Cross Research Ethics Committee reference 19/LO/0204). Written informed consent was obtained on enrolment to the trial and participants were asked to confirm their consent verbally prior to interview. Guidelines for reporting qualitative research were followed [21].

### Participants and recruitment

Interviewees were people living with myeloma undergoing ASCT who took part in the PERCEPT pilot trial. Purposeful sampling was used to select both intervention and control participants, approached in non-sequential order, across the trial period in order to capture the experiences of participants taking part at different times. Participants were asked to take part in an interview at or around the time of their final follow-up study assessment, approximately three months following ASCT and were approached by the lead author (OM) or research physiotherapist (JL).

### Qualitative interviews

The interviews were conducted using a semi-structured interview guide (Supplementary material). The interview schedule was developed by the research team and was designed to capture content related to experiences of ASCT, of being enrolled within a longitudinal study as part of the ASCT pathway, feelings and experiences of being randomised and perspectives on the study intervention and assessment processes. No predetermined theories or frameworks were used. Interviews were conducted by four researchers with training in qualitative research: the lead author, a female clinical academic physiotherapist (OM); a female research physiotherapist involved in delivery of the trial intervention (JL); and two (female and male) health psychology researchers (AR, GB). Interviews were conducted either face-to-face or by telephone depending on participant preference. The interviews were audio recorded, de-identified and transcribed verbatim by a professional transcription company with a UCL data sharing agreement.

### Analysis

Participant demographic data were collected from trial assessment forms completed at enrolment. Interview transcripts were analysed iteratively using the six phase process of reflexive thematic analysis (TA) [22]. Reflexive TA for this study was underpinned by the onto-epistemology of critical realism [23] using an approach that was both inductive and deductive.

Inductive codes were generated through repeated reading and assignment of coding labels to the text. Repeated rounds of analysis were carried out iteratively, revising and discarding codes and sets until the themes and subthemes were developed and described. Analysis was conducted by the first author (OM) with five (31%) of the transcripts double-coded by two other authors (AF, JL). These transcripts were discussed among the three researchers to confirm coding reliability.

In order to contribute to a greater theoretical understanding of elements of the trial intervention that contribute to trial participants’ engagement in it and potential behaviour change (i.e., in this case increased participation in exercise), deductive coding was used to identify components of the intended intervention discussed, both explicitly and implicitly, by intervention group participants. As this analysis related to the trial intervention, only those of the study sample who were members of the intervention arm and received the intervention during the trial were included in the deductive part of the analysis. Deductive codes were preconceived prior to analysis to identify content related to components of the exercise intervention including behaviour change techniques (BCTs) as defined by the BCT Taxonomy v1 [24]. A more positivist approach was used to draw out and describe the elements of the intervention that were discussed by quantifying the number of participants who mentioned each technique, and the frequency with which they appeared in the coded dataset. Qualitative data analysis was conducted using NVivo (version 12. QSR International Pty Ltd.) and descriptive statistics were analysed using Microsoft Excel.

## Results

Between November 2019 and June 2021, 33 participants completed the PERCEPT trial. From these, 16 participants (8 from each arm) were asked to participate in a qualitative interview and all approached participants (100%) agreed to be interviewed. Nearly all the interviewees underwent ASCT as first line treatment and had been diagnosed with myeloma in the region of six months prior to being approached for the trial. Approximately half of the interviewed participants had had significant spinal bone disease at diagnosis that required time immobilised with a spinal brace.

Data collection ceased at 16 interviews when the principle of saturation was reached and no new content relevant to the research questions were arising from interviews [25]. Six participants (38%), three from each study allocation group, were interviewed by the first author (OM). The physiotherapist who delivered the trial intervention (JL) interviewed four (25%) participants from the control group only. The remaining interviews were conducted by health psychology researchers (AR, GB) who were not involved in recruitment or delivery of the trial.

Interview length ranged from 23 minutes to 1 hour 14 minutes (mean duration 46 minutes). Mean interview duration was similar for intervention and control participants (Intervention: 47 minutes; Control: 46 minutes). The mean age of the study sample was 61 years (SD 11, range 37–72 years) and 9 (56%) were male (Table 1).

**Table 1:** Interviewee characteristics

Participant	Age at interview	Sex	Allocation
1	37	Female	Intervention
2	71	Female	Control
3	72	Male	Intervention
4	56	Male	Control
5	51	Female	Control
6	70	Male	Control
7	47	Female	Intervention
8	64	Male	Intervention
9	60	Male	Intervention
10	60	Female	Control
11	67	Male	Control
12	68	Female	Intervention
13	41	Male	Control
14	69	Female	Control
15	64	Male	Intervention
16	72	Male	Intervention

Four overarching themes (with ten subthemes) were identified. (1) “It’s not just beneficial for me, it’s for people after me as well”; (2) Disparities in experience of recovery – expectations, feeling prepared and support; (3) “What I wanted to do was build myself back up and prepare”; (4) Active ingredients – participants’ experience of the trial intervention. Themes and sub-themes are summarised in Table 2.

**Table 2:** Themes and related subthemes

	Theme	Subthemes
1	**“It’s not just beneficial for me, it’s for people after me as well”**	*Perception of participation as advantageous personally and altruistically*
		*Fortunate or disappointed – response to allocation*
		*Responses to allocation may indicate risk of contamination*
2	**Disparities in experience of recovery - expectations, feeling prepared and support**	*Discord in perceived ability or rate of return to ‘normal’ self*
		*Contrast between experiencing ongoing support and self-initiated support*
3	**“What I wanted to do was build myself back up and prepare”**	*Insufficient or restrictive advice regarding exercising with myeloma*
		*Exercise is preparation*
4	**Active ingredients – participants’ experience of the trial intervention**	*Exercise programme and sessions*
		*Behaviour Change Techniques (BCTs)*
		*Intervention Booklet*

### It’s not just beneficial for me, it’s for people after me as well

#### Perception of participation as advantageous personally and altruistically

Participants from intervention and control groups expressed similar reasons for taking part in the trial, principally that there was a perceived opportunity for personal gain in terms of physiotherapist input and support to exercise, as well as a perceived contribution to improving future supportive care for people living with myeloma.

*“I was hoping to be part of the group that actually got the extra exercise because I had such severe back issues with the myeloma. That was the first reason I wanted to do it but then beyond that, I just recognised that any additional physio we could get to get ourselves back to as much as a normal state as possible, could be beneficial” Participant 5, 51, female, Control*

Others described interest in participating as a way of ‘giving back’ for their myeloma medical treatment and a feeling of taking part as being worthwhile and potentially having an influence on future care for people living with myeloma undergoing ASCT.

#### Fortunate or disappointed – response to allocation

Most interviewees expressed clear desire for allocation to the intervention group. Nearly all control participants used the word ‘disappointed’ to describe the feeling they experienced when notified of their allocation. Some described a sense of loss of personal benefit from participation as a control but also acknowledged that they committed to the study and therefore continued participation. Most intervention participants expressed strong feelings of elation and relief at their allocation and the prospect of receiving the exercise intervention.

*“I can remember perfectly; it was relief. It was relief, I so wanted to be in the exercise, I so wanted to be in the exercise group and until I was told I was in the exercise group, it was like wishing for a lottery ticket [laughter]. So when I was told I was in the exercise group, I’d won the lottery.” Participant 8, 64, male, Intervention*.

In contrast to the controls some reflected that their continued participation in the study may not have occurred had they been allocated to the control group.

*“I was ecstatic, because I was told there were two parts of the study, and I was randomly chosen. And I did say to myself, if I’m not selected to be in the exercise, I don’t actually know if I’m going to do it.” Participant 1, 37, female, Intervention*.

A small number of participants expressed impartial views on their allocation and therefore were ambiguous about how they felt about it.

#### Responses to allocation may indicate risk of contamination

Interviewees shared narratives that may indicate possible risks of contamination of the control group. Participants were not blinded to their allocation and had not been asked to avoid discussion about their participation in the trial with others. Some intervention participants who found they were benefiting from the exercise intervention and regular support from the physiotherapist, reported sharing their positive experiences with other people living with myeloma treated within the centre and encouraging them to seek out support too.

*“And then I’d mentioned I speak to my physiotherapist. And he said, “Well, how did you get one of those?” And I told him what I was doing, that I was part of the study, and I asked him, ‘Was it offered to you?’.” Participant 1, 37, female, Intervention*.

Despite encouragement to continue their usual exercise behaviour during the trial period five control participants did report accessing additional physiotherapy support. These, and others, also described changes to their PA behaviour after enrolling in the study.

*“[Generic cancer physiotherapy service] were kind enough to see me and gave me an exercise handout and showed me a few of the myeloma exercises that I could do, and that was basically the replacement of not being able to be part of the exercise group… So once I had that information and all the exercises that I could do in the morning and the evening, I did start to do those exercises, which were of great help.” Participant 10, 60, female, Control*.

As well as indications of intentional access to physiotherapy support or exercise resources outside of the trial, some control participants also received input from physiotherapists during their ASCT admission if they spent time on the inpatient hospital ward as part of usual care.

### Disparities in experience of recovery – expectations, feeling prepared and support

#### Discord in perceived ability or rate of return to ‘normal’ self

There was universal acknowledgment by most intervention participants that their recovery was directly impacted by taking part in the trial intervention, with many indicating their recovery was quicker or less daunting than expected. Most intervention participants shared reflections on feeling a shift in their focus from a negative one related to undergoing intensive treatment with an assumed slow trajectory of recovery to a positive one, that their recovery was better than they expected and recalled less negative features of their recovery.

*“I think my recovery was probably better than I thought it would be because when you read about it you think it could be months recovery and it didn’t seem that bad after all.” Participant 16, 72, male, Intervention*.

Although there was common recall amongst all participants of the immediate consequences of ASCT treatment during admission (experiencing debilitating fatigue, gastrointestinal symptoms, nausea and reduced appetite), most intervention participants reported a trajectory of recovery that was at first slow but then ramped up quickly as they were able to build up their exercise through engagement with the physiotherapist. Most intervention participants reported feeling as physically recovered as they could possibly be and anticipated return to usual activities quicker than expected.

In contrast, control participants mostly recalled a consistently slow trajectory of recovery and at interview many reported not being anywhere near their pre-diagnosis function or abilities. Importantly, some control participants were explicit in their view that their expectations of returning to ‘normality’ were low or much further in the future. The concept of having control in one’s preparation for and recovery from ASCT was evident from how interviewees discussed their experiences of the process. Intervention interviewees were more optimistic and indicated active participation in determining their ongoing recovery, whereas controls were more passive in their discussions indicating a notion of waiting for things improve.

*“Now I can only do, at the moment, a quarter of the things that I would be able to do pre-cancer… But I’m hoping that, I’m going on to seven months after my transplant, that maybe towards the end of the year gradually things will get a bit better as well.” Participant 10, 60, female, Control*.

This acceptance of living with the ongoing consequences of their treatment was only evident among control participants. There was a sense that they were counselled to expect a recovery that would be challenging and slow and having started their ASCT treatment with lower physical capacity and ongoing symptom burden from induction treatment, they did not see themselves returning to their pre-diagnosis physical self any time soon. On the other hand, the intervention participants indicated their trajectory of recovery was nearly complete and related this to engaging in exercise before ASCT and being supported to recoup the benefits of their pre-ASCT activity through ongoing rehabilitative support following ASCT.

*“I’m so sure that there are going to be many more people like me that get this myeloma and have careers and things that they love to do that entail being physical. And if that’s who you are, then just knowing that it is possible to return to some level to that, it’s good for the soul. And if it’s good for the soul, it’s going to be good for your recovery.” Participant 8, 64, male, Intervention*.

#### Contrast between experiencing ongoing support and self-initiated support

Generally the interviewees reported positive experiences regarding support in the post-ASCT period although there was a notable contrast in access to and availability of specific support regarding their recovery. Control participants recalled receiving information about how to access urgent support via telephone should they require it and reported a sense of reassurance that contact with their clinical team was only a call away. Many control participants reflected on wanting more general support related to their physical recovery, and that although they could have called for advice or input, they often did not initiate a request for support because it was not urgent or an emergency. Some felt that they may have benefited from being checked on more regularly throughout their early recovery as opposed to needing to self-initiate input.

The intervention participants were collective in the value they placed upon the exercise and the regular individual contact they received from the study physiotherapist as having a role in making them feel physically prepared. They reflected that it was not purely the exercise programme but also the personalised approach to discussing what they were going through at each stage of treatment that was an instrumental part of their experience in trial. Some highlighted how discussing the challenges they were experiencing as well as monitoring and reporting their progress with the physiotherapist helped them to normalise their experiences and feel more optimistic about their recovery, focussing on problem solving, pacing their activities and progressing their tailored weekly exercise goals.

*“[Study physiotherapist] took me in hand and told me what she expected and what I had to do and it was just what I needed at that time. In fact, her phone calls regarding physiotherapy on the trial became a much more extended support to me.” Participant 12, 68, female, Intervention*.

There was a sense from interviews that whilst most controls felt they would have benefited from more support in the post-ASCT period, most could not be specific about what this would have involved. The intervention participants shared consistent reports of regular input from the physiotherapist to discuss what they were going through, whether specific to physical function and exercise or more related to emotional or practical recovery, was important in facilitating active participation in their own recovery as opposed to waiting for recovery to occur.

### What I wanted to do was build myself back up and prepare

#### Insufficient or restrictive advice regarding exercising with myeloma

Participants expressed a desire for advice and practical support specifically with regards to exercise prior to entering the trial. Most interviewees reported realising that engaging in exercise was required to counteract the resulting impact of their post-diagnosis inactivity. However, this was coupled with a profound sense of not knowing what exercise they could engage in and how to do so without advisory or practical support. Most were seeking more than general advice to keep active, desiring more individualised advice, personalised to how they could exercise during treatment. Nearly all reported receiving little or no advice on PA prior to the trial. Alarmingly, some interviewees recalled being told by health professionals to avoid exercise all together. Those who reported seeking out additional information found that they encountered inconsistent advice, that was in discord with previous guidance from their myeloma team or that was in conflict with what they believed about the importance of being active.

*“I did get referred to physio… she just kind of said ‘when you go in for your stem cell transplant, book something two months after your transplant and come back’, because they kind of were like ‘you’re not going to be able to do anything during your recovery from that.’ So it was a bit black and white, instead of ‘maybe you could do a little bit’… But should I have pushed myself a little bit more during the transplant or recovery? I’m not sure” Participant 5, 51, female, Control*.

Interviewees commonly shared their concern around uncertainty regarding the quantity or intensity of exercise they could engage in when exercising independently, and the lack of alternative support/guidance when dissatisfied with responses from health professionals.

There was greater dissatisfaction amongst the sample of interviewees who had had spinal bone disease. In contrast with the other interviewees, who had become inactive over the course of early treatment, but who were not specifically restricted in their function, interviewees who had experienced a period of spinal bracing shared experiences of rapid loss of strength, function and confidence due to immobilisation. They shared frustration at the ambiguity regarding how to be active safely, albeit with limitations, and demonstrated a greater appreciated need to become fitter because of how physically depleted they felt on removal of their brace and the prospect of their upcoming ASCT.

*“I understood it because they were worried about my skeleton. So I understood why the advice was, but the way I was feeling and I suppose because of my [profession] I’ve been taught to know and understand my body and what it feels like, and I suppose for me the restrictions were – I don’t know – it was too strong.” Participant 8, 64, male, Intervention*.

The desire for some to exercise was strong enough to result in them partaking in exercise despite advice not to, as they felt an ‘all or nothing’ approach was too restrictive.

*“Even though I’d had the cautionary advice from the hospital, I thought ‘Well, I can see that they’re saying these things because they don’t know and they have to say it because they don’t want to risk somebody like me ending up with a broken back or giving the wrong advice.’ But I did feel that it was overly cautious, so I did do things that I felt that my body could withstand without being stupid about it. I think my general sense of all the exercise advice I’ve received is that nobody really knows, and I’m as good as anybody else in making that assessment for myself.” Participant 4, 56, male, Control*.

#### Exercise is preparation

Participants frequently referred to the role of exercise or PA as important for their preparation for ASCT, as well as their future recovery. Most interviewees expressed a perception that they had wanted to physically prepare for their ASCT, for some it was the main motivation behind enrolling in the trial. Having recalled the negative effects of diagnosis and early treatment on their fitness and strength, nearly all reflected on the period around consideration for ASCT as a key time for contemplating engaging in exercise.

*“By the time I was recruited and I was looking forward to the stem cell transplant, I’d been through six months of initial therapy… I was looking forward to the stem cell transplant but very anxious and worried about the impact that would have, so I was thinking I need to get my body to be in as good shape as possible to be as strong as possible to withstand the onslaught of what was to come.” Participant 4, 56, male, Control*.

Interviewees did share concern that the loss of strength and fitness following diagnosis may have lowered their capacity to take on further intensive treatment. Therefore, getting fitter prior to ASCT was crucial to put them in a position where they could tolerate any further consequences of treatment.

*“They told me that the transplant would take a lot out of me, and in my mind there wasn’t much left to take out of me so I felt like I needed to do things to build up so that I had something to lose in the transplant stage.” Participant 8, 64, male, Intervention*.

Nearly all expressed feelings that getting fitter through exercise would have or did better equip them to manage their ASCT in the immediate acute phase and into recovery from treatment.

*“Oh being very weak, very weak and poorly. Yes, after the transplant for a while. I’m better now, but it’s taken a while to recover and I’m sure I would have recovered quicker had I been in better condition when it started.” Participant 6, 70, male, Control*.

For those who did receive the input from the physiotherapist and intervention exercise programme as part of the PERCEPT myeloma trial there was explicit appreciation for its positive influence on their ability to tolerate and recover from their ASCT.

*“I thought I’d go in, have it and bounce back. So it took me longer, but if I hadn’t got [study physiotherapist]’s support and the exercises, it would have taken twice as long. I don’t think I would have been fit enough to have the stem cell treatment.” Participant 12, 68, female, Intervention*.

Experiences shared by participants in the control group highlighted missed opportunities to support patients to prepare physically for upcoming treatment despite their desire for guidance. Several interviewees recalled being given advice to wait until after their transplant before initiating exercise, which was discordant with their perceived need to get into better physical condition for their upcoming intensive treatment. This was a common experience for the interviewees who had experienced spinal bracing and general lack of advice regarding exercising with myeloma-related bone disease.

*“I did ask them specifically… I had a telephone conversation with the [health professional] and I did go through my questions with her about what I could and couldn’t do… I remember her advice was cautionary and she said “I don’t really want you doing anything until you’ve got through the other end of the transplant.” Participant 4, 56, male, Control*.

### Active ingredients – participants’ experience of the trial intervention

#### Exercise programme and sessions

The role of the physiotherapist in providing regular input appeared to be the most valued part of the intervention for participants allocated to the intervention group, followed by the exercise programme itself. Overwhelmingly, interviewees valued the individualised, tailored approach to the programme made possible through their engagement with the physiotherapist. Some younger, previously active participants initially thought of the structured exercises specific to the trial as too basic but that with tailoring, they found a level that challenged them and saw progress.

*“I think the exercise given is geared towards people that are less mobile before. And so we kind of worked out something that worked well for me. But I think they were really decent exercises because they were strengthening exercises, which I would do anyway.” Participant 1, 37, female, Intervention*.

Other previously active participants also shared how they benefited most from the aerobic component of the programme and working with the physiotherapist to progress their fitness. Some interviewees felt they would have preferred more frequent supervised sessions, particularly pre-ASCT, in order to benefit from the higher intensity aerobic exercise they felt they achieved more easily through use of gym equipment and supervision of the physiotherapist. Other participants found the combination of resistance and strengthening exercises to be most impactful.

Collectively interviewees recalled how seeing their own progress over time spent following the exercise programme, whether through lifting more weight or completing more repetitions or through seeing changes to their body or fitness, was motivating and encouraged them to continue to adhere to the programme. A number of intervention participants reported to have continued elements of the programme as part of their ongoing exercise after they had completed the trial.

*“For me, the aerobic side of stuff was always challenging for me. The strength work, yes, in the beginning I suppose the exercises themselves were fine because all the exercises can be personalised to where I was, so everything that we did I’m still doing; I’m still doing that set of exercises because they work, it’s just that now I’m able to do the sets with weights and with the extra Pilates work that I do myself. Especially in the beginning, the programme was definitely enough and definitely worked all parts of the body.” Participant 8, 64, male, Intervention*.

#### Behaviour Change techniques (BCTs)

The trial intervention was informed by behaviour change theory and components of the intervention were mapped according to the BCT taxonomy (BCTT v1)[19, 24]. Previous themes already describe components of the intervention indicative of BCTs, such as ‘credible source’ evident from descriptions of education and guidance from a physiotherapist with expertise in myeloma. Additional BCTs were identified from the transcripts through implicit and explicit mentions by interviewees. The five most frequently coded BCTs were: (1) Goal-setting of the behaviour; (2) Graded tasks; (3) Adding objects to the environment; (4) Self-monitoring of behaviour; (5) Generalisation of behaviour.

The most common BCT referred to by most participants and mentioned most frequently throughout the transcripts was ‘goal-setting’. Most interviewees described positive experiences of setting and monitoring goals with the physiotherapist, with goals not only relating to exercise and PA behaviour but some described setting ‘lifestyle’ goals especially in the recovery or rehabilitation phase of the intervention. When talking about goal-setting most participants referred to the intervention booklet they received. Alongside mentions of the intervention booklet, the supply of heart-rate monitors and resistance exercise bands, as well as participants obtaining or using other exercise equipment were identified as the BCT ‘Adding objects to the environment’.

Intervention interviewees also explicitly referred to progression of the exercise programme, adaptations of exercises due to improvements in fitness and their progression in terms of ability to manage daily tasks in their recovery. These were mostly linked to discussion related to following the intervention programme and receiving support from the physiotherapist. These references were coded to the BCT ‘graded tasks’. Related to progression and referring frequently to use of the intervention booklet, participants stressed importance on ‘self-monitoring’ of exercise alongside recording goals. A small number of interviewees mentioned continued self-monitoring of their exercise behaviour using their own logs, based on the study log sheets. Shared examples included keeping a written notebook and a digital spreadsheet of continued activity beyond the trial period.

#### Intervention booklet

As previously described, most participants were candid in their regard for the intervention booklet as a key resource to support adherence to the intervention. There was suggestion that completing the log sheets brought about accountability to the intervention. Participants varied in their opinions of what part of the intervention booklet was most important for them. Many who discussed the importance of setting goals expressed having space in the intervention booklet to record their goals as valuable. Some reported recording adherence to the exercise and being able to look back at previous weeks to self-assess and monitor their progress as fundamental to their motivation, although others recalled that they did not complete these elements of the intervention booklet and described using it solely to refer to the specific instructions for the exercise programme. One participant referred to completing the log sheets as “more of a chore” and placed greater value on the weekly discussions with the physiotherapist for motivation.

## Discussion

This qualitative study investigated the experiences of people living with myeloma who took part in exercise related research whilst undergoing ASCT. The study focussed on gathering deeper understanding of participant engagement with trial processes as well as issues related to PA since diagnosis and during their participation in the trial. The themes generated from the coded dataset cumulated around three broad areas: common experiences related to participation in the trial irrespective of group allocation; contrasting experiences of participants related to allocation; and pre-enrolment experiences of living with myeloma, its early treatment and the effects on PA and the paucity of related support. An additional area developed described factors related to the intervention.

Reasons for participating in the exercise trial fell into both altruistic and personal motivations. Altruism is known to be an important influence in recruitment to health-related RCTs with evidence that trial participants can be motivated by a genuine wish to contribute to knowledge and improvement in care as well as a way of ‘giving back’ [26, 27]. Most of the interviewees expressed a personal motivation for participation with a perception of the trial intervention as potentially beneficial to them, therefore consistently those allocated to the control condition conveyed a sense of disappointment and dissatisfaction with their allocation. Acceptance of equipoise between allocation conditions requires a belief by participants that there is genuine uncertainty regarding the benefits of the intervention being investigated and that allocation to control may be as beneficial [28]. The indication of preference and possibility of negative feelings regarding allocation perhaps illustrates an unbalanced consideration and therefore deficient equipoise prior to participation and randomisation, both of which are considered important for ethical, informed consent in trials [29–31].

Testing complex interventions, such as rehabilitation and behavioural interventions, through traditional RCT designs is inherently undermined by an inability, in most cases, to blind participants to their allocation [32]. The use of usual care as the control comparator in the PERCEPT trial was determined to be appropriate given that there was no standardised approach to offering physiotherapy or exercise therapy to people living with myeloma. However, this study highlights how approached and enrolled participants had preconceived perceptions of benefit to be gained from the intervention. Interviewees associated potential benefit from taking part purely because of the physical and functional deficits they had experienced and lack of advice and ameliorative support they had been able to access since diagnosis. Other studies have documented that participant dissatisfaction with their current status or a dearth of acceptable standard of usual care drives motivation to participate in trials [31]. One exploration from a stroke rehabilitation pilot trial described participants ‘desperation’ to do anything to help their situation as a personal motivator to take part, but this was met with feelings of ‘abandonment’ when allocated to an inactive control condition [28]. Another qualitative study embedded within a rehabilitation pilot trial reported people with colorectal cancer allocated to a no rehabilitation control group felt abandonment also [33]. The possibility of participation in the trial as potentially beneficial and probable lack of balanced consideration for outcome of allocation may have instilled therapeutic expectations within participants, quickly rescinded when informed of their allocation to control.

Actions of both control and intervention participants interviewed for this qualitative study identified areas for concern regarding the fidelity of the control condition. Dissatisfaction from allocation to control may not only lead to negative feelings but has been seen to induce dropout and increase attrition bias in RCTs [34, 35], although this was not evident in this trial [20]. Most control group interviewees reflected a shift from disappointment because of their primarily personal motivation to take part towards a more altruistic stance that their participation was still important to benefit the research and therefore remained in the trial. Although their dissatisfaction may not have played out as attrition it did result in many seeking alternatives to the intervention outside of the trial.

Contamination bias arises when the control group participants are inadvertently exposed to or receive the intervention condition and can result in muted or completely masked effects of the intervention being trialled [36, 37]. A systematic review of contamination, dropout and control group design in exercise oncology trials found 37% of trials reported contamination of control group [38]. It is likely that this pilot trial was exposed to contamination bias in two ways. Firstly, more than half of control participants interviewed reported seeking out physiotherapy support or independently becoming more physically active during the trial period therefore it is possible that this also occurred in other control group participants in the wider trial. Secondly, intervention participants, so pleased with their experience of the trial intervention and engagement with the physiotherapist, reported encouraging other patients to seek out or request referral for physiotherapy. This informal proclamation of benefit by the intervention group could be considered a source of contamination if directed towards control participants or other patients who were later approached for the trial [36].

It has been established that perceived personal gain comes before altruism as motivating factors in trial participation, but it has also been proposed that altruism becomes the primary motivator for control participants after randomisation has occurred [39]. As people are often motivated primarily at a personal level, future trial designs where blinding of allocation is not possible may need to consider alternative methods to bring about equipoise to randomisation and reduce dissatisfaction with allocation. Improved recruitment and retention, as well as reduced contamination of control could be facilitated by using alternative designs including double consent processes such as Zelen design [40–43] or patient preference trial designs [6], as well as offering control conditions that could be perceived to be as advantageous as the intervention condition.

Although there may be indications of research participation effects, contamination bias and potential deleterious impact on trial outcomes due to participation of controls in exercise or physiotherapy, what became evident from this qualitative study is that there remained polarised experiences of physical and emotional recovery from ASCT consistent with allocation. Most control participants described both an expectation and reality of a slowly progressing recovery that they expected to go beyond the trial period, whereas intervention participants were applauding of their rate of recovery and attributed it to the intervention, in particular the regularity of support from the physiotherapist. It has been hypothesised that participation within research procedures alone may induce the behaviour change under investigation [5, 7]. However, in the case of these interviewees the probable influence of participation on eliciting some form of change in PA behaviour before ASCT may not have been enough to impact their trajectory of recovery following ASCT. These findings complement trial results indicating the intervention induced possible benefit and may influence recovery following treatment as intensive as ASCT [20] but group differences may have been impacted by contamination. However, benefits seen may relate as much to the psychological, emotional and cognitive effects of the intervention as the physiological.

What components of the PERCEPT trial intervention were responsible for these perceived outcomes was also explored. Intervention participants placed great importance upon the regular contact with the physiotherapist and although these contacts were related to intended intervention mechanisms (e.g. BCTs such as goal setting, graded tasks) that would be facilitated through discussion with the physiotherapist, this could also have been related to the additional attention received. Individualised support from a physiotherapist or other exercise specialist, as well as goal-setting have also been reported as a motivator to PA engagement in qualitative explorations among solid oncology patients [44, 45]. The additional attention received through intervention delivery alone may be sufficient to induce change in participants receiving behavioural interventions [46]. The lack of additional attention afforded to the control group may pose a confounding factor in the trial. In retrospect, little consideration was given to the general therapeutic factors at play within the intervention group that may have been necessary to provide and therefore control for in the control condition [46, 47]. It is therefore difficult to tease out whether it was the expected mechanisms of the exercise programme or regular therapeutic engagement with the physiotherapist, or both, that influenced intervention participants experience of recovery as a more positive one than that experienced by the control participants.

Analysis of intervention participants’ experiences of undergoing the exercise intervention did provide insight into the possible ‘active ingredients’ or mechanisms at play. Explicit references were made to the structured exercise prescribed despite overall greater significance was placed upon the physiotherapist contact, particularly in the post-ASCT rehabilitation phase. Intervention materials, particularly the intervention booklet were also highlighted as central to their engagement in the intervention. Descriptions of participation in the intervention activities did provide evidence for the BCTs at play. The most frequently coded BCTs from this qualitative study include those associated with interventions that support long-term PA behaviour change in cancer survivors [48]. Interviewees placed importance on goal-setting and grading of tasks facilitated by the physiotherapist using an individualised approach. The benefits of practitioner support with goal-setting, setting of graded tasks as well as reviewing progress and self-monitoring of behaviour have been highlighted as influential in other exercise interventions [49, 50]. The BCTs reported within this qualitative study are in line with those reported in other studies, confirming these elements of the intervention as likely mechanisms of influence on exercise behaviour.

Another important finding from this study is the clear desire from interviewees to use the phase in their treatment trajectory, between commencement of induction chemotherapy and ASCT, as a period of physical restoration and preparation. Most significantly, is the commonality of experience that seeking support or practical advice related to physical conditioning in this phase of treatment is most often met with unsatisfactory response. Poor physical functioning and perceived loss of control are associated with worse psychosocial outcomes and quality of life in people undergoing stem cell transplantation [51, 52]. Qualitative findings among people undergoing allogeneic transplantation highlight that exercise is perceived as positively influencing recovery from treatment and can provide a sense of control and structure during transplant [53]. Transplant recipients welcomed support from clinicians, resources to support exercise and measurement of physical outcomes and that these provided automatic incentives and motivation to exercise during transplant [53]. Other qualitative literature specifically amongst myeloma ASCT recipients have also found that patients place importance on the role of exercise for enhancing recovery [11, 12, 54, 55]. Therefore, the finding that people living with myeloma preparing for ASCT were seeking of PA advice to exercise some control in their recovery is expected. However, the experience of most people interviewed in this study is that there is little or no access to specialist advice or individually tailored support that people living with myeloma regard as necessary to support their confident, safe-engagement in PA. Participants reporting a motivation to exercise to ‘build back up’ following early consequences of treatment and ‘prepare’ for upcoming ASCT but being met with lacking or inconsistent advice, indicates an obvious missed opportunity to fulfil a clear patient initiated desire to self-manage in their treatment journey. This study adds further support for embedding provision of exercise and rehabilitation support within the care pathway for myeloma, particularly in preparation for ASCT [8, 12].

### Limitations

There is no doubt that the addition of qualitative investigation alongside or embedded within RCTs is an essential component to understanding the intricacies of developing and testing complex interventions [1, 3, 4, 31], however this research is not without limitation. The role of the lead researcher and their perspectives as a rehabilitation professional will have shaped the research analysis. This acknowledgment of the role of the researcher in the research process, use of reflexive thematic analysis as well as the inclusion of other researchers to interview and code a selection of transcripts will have contributed to reliability. In addition, although the study sample was purposefully sampled to gather experiences of participants recruited across the length of the trial recruitment period, including those who were recruited to both the original face-to-face and virtual study protocols, the interview schedule was not adapted to explore experiences of the different protocols or the impact of the COVID-19 pandemic on participation. Therefore, this study may have missed opportunities to provide further context to the different modes of trial delivery.

This qualitative study has highlighted possible research participation effects that could occur both pre- and post-randomisation to undermine the outcome of intervention effects and will need to be considered in the design of future research in this area. It is evident from literature that the population from which the trial was sampled were likely to be motivated and contemplative of seeking out support to exercise. Regardless of their state of readiness or capability to independently engage in more PA, it is possible that being provided with information related to the exercise trial and undergoing trial procedures may have been sufficient for some to change their PA behaviour as well as posing a challenge to the concept of equipoise required for randomisation.

## Conclusions

This qualitative study investigated the experiences of participation in the PERCEPT myeloma RCT, which compared an exercise intervention with incorporated BCTs delivered before, during and after ASCT for people living with myeloma, compared to usual care. The findings indicate that study participants held common beliefs regarding lack of reliable, responsive PA advice and that participation in an exercise intervention prior to ASCT would be advantageous. There were contrasting views related to allocation, with disappointment evident from control participants which may have led to contamination of control condition. In addition, intervention participants described factors related to the intervention that were of importance, most commonly the role of the physiotherapist and goal-setting.

This study aligns and adds to qualitative literature exploring the experiences of people living with myeloma undergoing ASCT treatment and their desire for support to exercise. The phase in their treatment, between early induction treatment and decision to proceed with ASCT represents a key time of contemplation for people living with myeloma considering becoming more active and wishing to physically optimise themselves for future treatment. Findings from this embedded qualitative study indicate considerations required when designing pilot and efficacy trials of complex interventions, for which multi- or mixed-methods research is likely to provide nuanced insight into motivations for participation, intervention mechanisms at play as well as effects of participation that may impact interpretation of quantitative outcomes.

## Electronic supplementary material

Below is the link to the electronic supplementary material.

Supplementary Material 1

## Data Availability

The data that support the findings of this study are available from the corresponding author, upon reasonable request.

## List of abbreviations

RCT: randomised controlled trial
BCTs: behaviour change techniques
ASCT: autologous stem cell transplant
PA: physical activity
TA: thematic analysis

## References

[1] Skivington K, Matthews L, Simpson SA, Craig P, Baird J, Blazeby JM (2021). A new framework for developing and evaluating complex interventions: update of Medical Research Council guidance. BMJ.

[2] Oakley A, Strange V, Bonell C, Allen E, Stephenson J, Team RS (2006). Process evaluation in randomised controlled trials of complex interventions. BMJ.

[3] Lewin S, Glenton C, Oxman AD. Use of qualitative methods alongside randomised controlled trials of complex healthcare interventions: methodological study.Bmj-British Medical Journal. 2009;339. 10.1136/bmj.b3496 PMC2741564 19744976

[4] Baldeh T, MacDonald T, Kosa SD, Lawson DO, Stalteri R, Olaiya OR (2020). More pilot trials could plan to use qualitative data: a meta-epidemiological study. Pilot Feasibility Stud.

[5] McCambridge J, Kypri K, Elbourne D (2014). Research participation effects: a skeleton in the methodological cupboard. J Clin Epidemiol.

[6] King M, Nazareth I, Lampe F, Bower P, Chandler M, Morou M (2005). Impact of participant and physician intervention preferences on randomized trials: a systematic review. JAMA.

[7] McCambridge J, Kypri K, Elbourne D (2014). In randomization we trust? There are overlooked problems in experimenting with people in behavioral intervention trials. J Clin Epidemiol.

[8] Snowden JA, Greenfield DM, Bird JM, Boland E, Bowcock S, Fisher A (2017). Guidelines for screening and management of late and long-term consequences of myeloma and its treatment. Br J Haematol.

[9] Gozzetti A, Candi V, Papini G, Bocchia M (2014). Therapeutic advancements in multiple myeloma. Front Oncol.

[10] Wang XS, Shi Q, Williams LA, Shah ND, Mendoza TR, Cohen EN (2015). Longitudinal analysis of patient-reported symptoms post-autologous stem cell transplant and their relationship to inflammation in patients with multiple myeloma. Leuk Lymphoma.

[11] Craike MJ, Hose K, Courneya KS, Harrison SJ, Livingston PM (2013). Perceived benefits and barriers to exercise for recently treated patients with multiple myeloma: a qualitative study. BMC Cancer.

[12] Walpole G, Clark H, Dowling M (2018). Myeloma patients’ experiences of haematopoietic stem cell transplant: a qualitative thematic synthesis. Eur J Oncol Nurs.

[13] Servadio M, Cottone F, Sommer K, Oerlemans S, van de Poll-Franse L, Efficace F. Physical activity and health-related quality of life in multiple myeloma survivors: the PROFILES registry.BMJ Support Palliat Care. 2019. 10.1136/bmjspcare-2018-001755 31253733

[14] Jones LW, Courneya KS, Vallance JK, Ladha AB, Mant MJ, Belch AR (2004). Association between exercise and quality of life in multiple myeloma cancer survivors. Support Care Cancer.

[15] Smith L, McCourt O, Henrich M, Paton B, Yong K, Wardle J et al. Multiple myeloma and physical activity: a scoping review.BMJ Open. 2015;5(11). 10.1136/bmjopen-2015-009576 PMC4663409 26614625

[16] Gan JH, Sim CYL, Santorelli LA (2016). The effectiveness of exercise programmes in patients with multiple myeloma: a literature review. Crit Rev Oncol/Hematol.

[17] Nicol JL, Chong JE, McQuilten ZK, Mollee P, Hill MM, Skinner TL (2023). Safety, Feasibility, and efficacy of Exercise Interventions for people with multiple myeloma: a systematic review. Clin Lymphoma Myeloma Leuk.

[18] McCourt O, Fisher A, Ramdharry G, Roberts AL, Land J, Rabin N (2022). Adaptation of the PERCEPT myeloma prehabilitation trial to virtual delivery: changes in response to the COVID-19 pandemic. BMJ Open.

[19] McCourt O, Fisher A, Ramdharry G, Roberts AL, Land J, Rabin N (2020). PERCEPT myeloma: a protocol for a pilot randomised controlled trial of exercise prehabilitation before and during autologous stem cell transplantation in patients with multiple myeloma. BMJ Open.

[20] McCourt O, Fisher A, Ramdharry G, Land J, Roberts AL, Rabin N et al. Exercise prehabilitation for people with myeloma undergoing autologous stem cell transplantation: results from PERCEPT pilot randomised controlled trial.Acta Oncologica. 2023. 10.1080/0284186X.2023.2178326 36794394

[21] O’Brien BC, Harris IB, Beckman TJ, Reed DA, Cook DA (2014). Standards for reporting qualitative research: a synthesis of recommendations. Acad Med.

[22] Braun V, Clarke V, Clarke V (2013). Successful qualitative research: a practical guide for beginners / Virginia Braun & Victoria Clarke. Los Angeles.

[23] Mukumbang FC. Retroductive Theorizing: A Contribution of Critical Realism to Mixed Methods Research.J Mix Method Res. 2021.

[24] Michie S, Richardson M, Johnston M, Abraham C, Francis J, Hardeman W (2013). The behavior change technique taxonomy (v1) of 93 hierarchically clustered techniques: building an international consensus for the reporting of behavior change interventions. Ann Behav Med.

[25] Saunders B, Sim J, Kingstone T, Baker S, Waterfield J, Bartlam B (2018). Saturation in qualitative research: exploring its conceptualization and operationalization. Qual Quant.

[26] Houghton C, Dowling M, Meskell P, Hunter A, Gardner H, Conway A (2020). Factors that impact on recruitment to randomised trials in health care: a qualitative evidence synthesis. Cochrane Database Syst Rev.

[27] Land J, Hackett J, Sidhu G, Heinrich M, McCourt O, Yong KL et al. Myeloma patients’ experiences of a supervised physical activity programme: a qualitative study.Support Care Cancer. 2022. 10.1007/s00520-022-07062-x PMC9035778 35467117

[28] Norris M, Poltawski L, Calitri R, Shepherd AI, Dean SG, ReTrain T (2019). Hope and despair: a qualitative exploration of the experiences and impact of trial processes in a rehabilitation trial. Trials.

[29] Miller FG, Brody H (2003). A critique of clinical equipoise. Therapeutic misconception in the ethics of clinical trials. Hastings Cent Rep.

[30] Rooshenas L, Elliott D, Wade J, Jepson M, Paramasivan S, Strong S et al. Conveying Equipoise during Recruitment for Clinical Trials: Qualitative Synthesis of Clinicians’ Practices across Six Randomised Controlled Trials.Plos Medicine. 2016;13(10). 10.1371/journal.pmed.1002147 PMC5068710 27755555

[31] Toye F, Williamson E, Williams MA, Fairbank J, Lamb SE (2016). What value can qualitative research add to quantitative Research Design? An Example from an adolescent idiopathic scoliosis trial feasibility study. Qual Health Res.

[32] Boutron I, Tubach F, Giraudeau B, Ravaud P (2004). Blinding was judged more difficult to achieve and maintain in nonpharmacologic than pharmacologic trials. J Clin Epidemiol.

[33] Hubbard G, O’Carroll R, Munro J, Mutrie N, Haw S, Mason H (2016). The feasibility and acceptability of trial procedures for a pragmatic randomised controlled trial of a structured physical activity intervention for people diagnosed with colorectal cancer: findings from a pilot trial of cardiac rehabilitation versus usual care (no rehabilitation) with an embedded qualitative study. Pilot Feasibility Stud.

[34] Higgins JP, Altman DG, Gotzsche PC, Juni P, Moher D, Oxman AD (2011). The Cochrane collaboration’s tool for assessing risk of bias in randomised trials. BMJ.

[35] Lindstrom D, Sundberg-Petersson I, Adami J, Tonnesen H (2010). Disappointment and drop-out rate after being allocated to control group in a smoking cessation trial. Contemp Clin Trials.

[36] Levin KA (2005). Study design II. Issues of chance, bias, confounding and contamination. Evid Based Dent.

[37] Robinson K, Allen F, Darby J, Fox C, Gordon AL, Horne JC (2020). Contamination in complex healthcare trials: the falls in care homes (FinCH) study experience. BMC Med Res Methodol.

[38] Steins Bisschop CN, Courneya KS, Velthuis MJ, Monninkhof EM, Jones LW, Friedenreich C (2015). Control group design, contamination and drop-out in exercise oncology trials: a systematic review. PLoS ONE.

[39] Harrop E, Noble S, Edwards M, Sivell S, Moore B, Nelson A et al. “I didn’t really understand it, I just thought it’d help”: exploring the motivations, understandings and experiences of patients with advanced lung cancer participating in a non-placebo clinical IMP trial.Trials. 2016;17. 10.1186/s13063-016-1460-8 PMC4955155 27439472

[40] Adamson J, Cockayne S, Puffer S, Torgerson DJ (2006). Review of randomised trials using the post-randomised consent (Zelen’s) design. Contemp Clin Trials.

[41] Land J, McCourt O, Heinrich M, Beeken RJ, Koutoukidis DA, Paton B (2020). The adapted Zelen was a feasible design to trial exercise in myeloma survivors. J Clin Epidemiol.

[42] Zelen M (1979). A new design for randomized clinical trials. N Engl J Med.

[43] Koutoukidis DA, Land J, Hackshaw A, Heinrich M, McCourt O, Beeken RJ et al. Fatigue, quality of life and physical fitness following an exercise intervention in multiple myeloma survivors (MASCOT): an exploratory randomised Phase 2 trial utilising a modified Zelen design.Br J Cancer. 2020. 10.1038/s41416-020-0866-y PMC7374110 32435057

[44] Ferri A, Gane EM, Smith MD, Pinkham EP, Gomersall SR, Johnston V (2021). Experiences of people with cancer who have participated in a hospital-based exercise program: a qualitative study. Support Care Cancer.

[45] Avancini A, Tregnago D, Rigatti L, Sartori G, Yang L, Trestini I et al. Factors Influencing Physical Activity in Cancer Patients During Oncological Treatments: A Qualitative Study.Integrative Cancer Therapies. 2020;19. 10.1177/1534735420971365 PMC7758643 33349064

[46] LaFave SE, Granbom M, Cudjoe TKM, Gottsch A, Shorb G, Szanton SL (2019). Attention control group activities and perceived benefit in a trial of a behavioral intervention for older adults. Res Nurs Health.

[47] Safer DL, Hugo EM (2006). Designing a control for a behavioral group therapy. Behav Ther.

[48] Grimmett C, Corbett T, Brunet J, Shepherd J, Pinto BM, May CR (2019). Systematic review and meta-analysis of maintenance of physical activity behaviour change in cancer survivors. Int J Behav Nutr Phys Act.

[49] Turner RR, Steed L, Quirk H, Greasley RU, Saxton JM, Taylor SJ (2018). Interventions for promoting habitual exercise in people living with and beyond cancer. Cochrane Database Syst Rev.

[50] McAuliffe H, Mc Sharry J, Dunne D, Byrne M, Meade O (2021). Identifying the active ingredients of cardiac rehabilitation: a behaviour change technique and qualitative analysis. Br J Health Psychol.

[51] Pulewka K, Wolff D, Herzberg PY, Greinix H, Heussner P, Mumm FHA (2017). Physical and psychosocial aspects of adolescent and young adults after allogeneic hematopoietic stem-cell transplantation: results from a prospective multicenter trial. J Cancer Res Clin Oncol.

[52] Coolbrandt A, Grypdonck MHF (2010). Keeping courage during stem cell transplantation: a qualitative research. Eur J Oncol Nurs.

[53] Abo S, Parry SM, Ritchie D, Sgro G, Truong D, Denehy L (2022). Exercise in allogeneic bone marrow transplantation: a qualitative representation of the patient perspective. Support Care Cancer.

[54] Craike M, Hose K, Courneya KS, Harrison SJ, Livingston PM (2017). Physical activity preferences for people living with multiple myeloma: a qualitative study. Cancer Nurs.

[55] Coon SK, Coleman EA (2004). Exercise decisions within the context of multiple myeloma, transplant, and fatigue. Cancer Nurs.

