# Supplementary material for: “What I wanted to do was build myself back up and prepare”: qualitative findings from the PERCEPT trial of prehabilitation during autologous stem cell transplantation in myeloma
Source: BMC Cancer. 2023 Apr 17;23:348. doi: 10.1186/s12885-023-10799-1 (PMC10107576; doi:10.1186/s12885-023-10799-1)
Supplement: Supplementary file 1 — Supplementary Material 1 [file 12885_2023_10799_MOESM1_ESM.pdf]

Myeloma Transplant Exercise Study
Interview Schedule – T3 Trial Participants

This guide is to ensure key aspects are covered during the interview. However a respondent-sensitive approach should be taken, allowing deviation from question order and raising additional issues if desired.

Interviewers should be mindful of the sensitive subject of the interview.

Introduction
Introduce yourself and explain purpose of the interview: to explore the participant's experience of the myeloma intervention and their experience of being a participant in the trial. Ask permission to record the conversation. Assure confidentiality and ask participant to be as open and honest as they can be.

Motivational factors

- What was your main reason for taking part in this research?
  - *Probe:* to improve wellbeing or fitness/to help contribute to research/to receive additional monitoring by Health Professionals?

Demotivating factors

- Was there anything that made you think twice about taking part in the research?
  - *Probe*: personal concerns - practicalities/factors relating to your illness or stem cell transplant treatment?
  - *Probe*: Trial concerns - factors relating to the research itself?

General trial feedback

- How did you feel about being asked to take part in this research initially, i.e. on referral for stem cell transplant treatment

- How did you feel when you were informed of your allocation to … group?
  - *Prompt:* pleased/confused/surprised/annoyed/excited

Physical Activity Prior to and during Myeloma Treatment

- Can you tell me about your physical activity levels before you started your treatment for myeloma and how this has changed during treatment?
  - *Probe*: occupational/recreational/sporting activities
  - *Probe:* How often, how long
  - *Probe:* What caused the person to change, if change mentioned

- Did you receive any advice or support to maintain your physical activity when you started your treatment for myeloma?
  - *Probe:* from whom? What advice? Did you follow it?

- Thinking specifically about preparing for your stem cell transplant. Did you receive any information, other than from the researchers, about physical activity or exercise while undergoing a transplant?
  - *Probe:* Did the Clinical Nurse Specialists or doctors provide support and information?
  - *Probe:* Did you seek or look for information?

Usual Care Group Participant:

- You have already spoken about how you felt about your allocation (above), now can you tell me about your physical activity behaviour after enrolling in the study and learning you were in the usual care group?

- How did you feel physically during your transplant admission and did you require any rehabilitation or physical support in hospital?
    - *Probe:* did your symptoms affect your daily routine or function?
    - *Probe:* How did what you described affect you, how did you feel?

- How has your recovery been since leaving hospital after your transplant?
    - *Probe*: levels of symptoms/function/physical activity
    - *Probe:* accessing support/advice/input for any concerns raised

Exercise Group Participant:

- Before your transplant, was the travel to your gym sessions ok?

- How did you feel about the frequency of gym sessions?
    - *Prompt*: was it useful to attend the gym as often as you did? Or were the session too frequent for your liking?
      if so how often would you have liked to have them?

- What did you think of the exercise you were prescribed?
    - *Probe:* did you feel they were either too arduous, or not arduous enough?
      did you have any concerns or worries?

- Were you able to carry out the prescribed exercises ok in the gym?

- How about in hospital?

- How about at home?
    - *Probe:* Were you able to exercise to the same intensity/level as in the gym?
    - If not, why do you think this would be?

- When you weren't able to carry out the exercises at any stage, why was this?

- How did you feel about the level of contact you had with the Physio during your hospital admission and after discharge?
    - *Prompt:* would you have liked any more or less contact? If so why?

- How did doing the exercise make you feel while you were awaiting your transplant?

- How did doing the exercise make you feel on the whole?
    - *Prompt:* enjoyable/happy/energised/tired/increased levels of pain

- How did you find being in a group with other Myeloma patients, when in the gym?
    - *Prompt:* did it result in extra support/reduce contact time with the Physio?

- What did you think of the behaviour change support provided?

- *Probe:* have you used any of them?

- How useful would you say the behaviour change support was in terms of helping you change your behaviours?
    - *Probe:* which elements were most helpful. E.g. log book, goal setting, rewards?
      which were least helpful?

- What did you think of the log books?
    - *Probe:* did you fill it out?
      did you use it to reflect on how you'd done over the weeks?
      was there anything about it you would change?

- Overall, do you think exercising and having support to be active before and during your transplant affected your recovery after discharge?
    - *Probe:* What do you think was most important? How did it make you feel?
    - *Probe:* Was the recovery period after hospital what you expected?

Assessments

- How were the assessments?
    - How did you find the length of assessments?
    - What did you think of the questionnaires?

- Were you happy with the tests and measures that were carried out (e.g. walking test, muscle strength, wearing the accelerometer?)
    - *Probe:* was there anything you felt should have been done differently?
      Was there anything you were uncomfortable with?

- How did you perceive the skills and knowledge of the researcher/s who saw you for your baseline and follow-up assessments?
    - *Probe:* did you like their approach?
      how was their manner?

General

- Can you tell me about the support you received before and during and after your transplant?
    - From health professionals
    - From family/friends
    - *Prompt:* was the support practical, emotional or both?
    - *Probe:* if yes, how important was that to you?
      if no, do you think this could have helped?

- Can you tell me how you felt, overall, about being a participant in this research?
    - *Probe:* what were the benefits of taking part?
      What were the disadvantages?

- Do you think that you have learned anything as a result of taking part in the trial?
    - *Probe:* do you think you have gained anything?

- Would you recommend taking part to other people who have myeloma or are having a transplant?

- Is there anything you think we should have done differently?

- Is there anything else you would like to say about the trial, that we’ve not already talked about?

Concluding comments and thanks
Thank the participant for their time today and ongoing and let them know where they can contact you in the future if they do have any additional comments.
